# Patterns of sex-biased dispersal are consistent with social and ecological constraints in a group-living cichlid fish

**DOI:** 10.1186/s12862-022-01980-4

**Published:** 2022-03-02

**Authors:** Aneesh P. H. Bose, Lukas Koch, Johanna Dabernig-Heinz, Jacqueline Grimm, Kristina M. Sefc, Alex Jordan

**Affiliations:** 1 Department of Collective Behaviour, Max Planck Institute of Animal Behavior, Konstanz, Germany; 2 Centre for the Advanced Study of Collective Behaviour, University of Konstanz, Konstanz, Germany; 3 Department of Biology, University of Konstanz, Konstanz, Germany; 4 Institute of Biology, University of Graz, Graz, Austria

**Keywords:** Relatedness estimators, Microsatellite genotyping, African cichlid, Habitat saturation, Cooperation, Social conflict, Population structure, Ecological constraints

## Abstract

**Background:**

Sex-biased dispersal is a common and widespread phenomenon that can fundamentally shape the genetic structure of the social environments in which animals live. For animals that live in and move between social groups, sex-biased dispersal can result in an asymmetry in the degree of relatedness among cohabiting males and females, which can have strong implications for their social evolution. In this study, we measured the relatedness structure within and across groups of a wild population of *Neolamprologus multifasciatus*, a highly-social, shell-dwelling cichlid fish endemic to Lake Tanganyika, East Africa. In total, we genotyped 812 fish from 128 social groups at 20 microsatellite loci. *Neolamprologus multifasciatus* live at high densities, and also experience strong ecological constraints on free movement throughout their habitat. At the same time, they exhibit sex differences in the degree of reproductive competition within their groups and this makes them an excellent model system for studying the factors associated with sex-biased dispersal.

**Results:**

Social groups of *N. multifasciatus* consist of multiple males and females living together. We found that cohabiting females were unrelated to one another (Lynch-Ritland estimates of relatedness = 0.045 ± 0.15, average ± SD), while males shared much higher, albeit variable, levels of relatedness to other males in their groups (0.23 ± 0.27). We uncovered a pronounced decline in relatedness between males living in separate groups as the spatial separation between them increased, a pattern that was not evident in females. Female dispersal was also markedly constrained by the distribution and availability of nearby territories to which they could emigrate.

**Conclusions:**

Our results indicate female-biased dispersal in *N. multifasciatus.* Our study also highlights how the spatial distribution of suitable dispersal destinations can influence the movement decisions of animals. We also emphasize how sex-biased dispersal can influence the relatedness structure of the social environment in which individuals interact and compete with one another.

**Supplementary Information:**

The online version contains supplementary material available at 10.1186/s12862-022-01980-4.

## Introduction

Dispersal is a ubiquitous life history trait that, through the mixing of individuals, influences the genetic structure of animal groups and populations, setting the social context in which selection operates [1, 2]. Dispersal decisions are thought to be part of a strategy that improves the breeding conditions of individuals, and can generally be categorized into natal dispersal, i.e., the movement of individuals from their birthplaces to the locations of their first breeding, and breeding dispersal, i.e., the movement of individuals between consecutive reproductive attempts [1, 3, 4]. For many organisms, dispersal is not uninhibited, but is rather influenced by their dispersal capacity and the availability of suitable patches on which to settle that are within reachable distances [5]. For example, in Mauritius kestrels, *Falco punctatus*, females typically disperse within 2 km of their natal patch, but can be forced to disperse over much farther distances when local breeding sites are limiting [6]. Also, in the spotted hyena, *Crocuta crocuta*, male dispersal patterns are dictated by the distribution of available clans to join and their relative qualities [7]. In general, dispersal is thought to be variable and plastic, with decisions about when to disperse, how far to disperse, and where to settle differing among individuals and across different environmental circumstances [2, 5]. Therefore, in order to better understand the forces that shape the genetic structure of populations, groups, and their social environments, it is important to determine which individuals disperse, what factors determine their dispersal behaviour, and whether these factors affect certain individuals disproportionately to others.

Dispersal frequently differs between males and females of a species with one sex dispersing more readily and over greater distances than the other [8]. For social animals that live in discrete groups, sex biases in the propensity for individuals to emigrate will shape the relatedness structure of their groups, and therefore also the social arena in which individuals interact and compete with one another. Sex-biased dispersal can cause one sex to be more closely related to their same-sex group mates than the other, resulting in asymmetric distributions of relatedness between interacting males and females and sex differences in selection for behavioral strategies. Sex-biased dispersal can therefore have far-reaching consequences for the evolution and expression of cooperative and social behaviours [9–12] and is fundamental to our understanding of social evolution [13].

Male-biased dispersal is particularly common in mammals (e.g., common voles, *Microtus arvalis*, [14]), while female-biased dispersal is typically more prevalent in birds (e.g., Seychelles warblers, *Acrocephalus sechellensis*, [15]). The current body of work on sex-biased dispersal suggests that selection can result in drastically different dispersal strategies for each sex [8, 16], and a myriad of ecological and social factors have been put forward in an attempt to explain these sex-specific patterns in nature. For example, sex biases in dispersal can arise when one sex experiences more intense local mate or resource competition [17–19], greater risk of inbreeding [19–22], or more pronounced constraints on emigration and settlement than the other [16, 23]. Many of the existing hypotheses for the evolution of sex-biased dispersal stem from a wealth of early work that focused on mammals and birds [8, 17, 24], but examinations of sex biases in dispersal have been increasing for other taxa as well. Fishes offer intriguing systems in which to study sex-biased dispersal because they display tremendous variation in mating systems, sociality, territorial behaviour, sex determination mechanisms, and parental care strategies [25–28], all factors that could affect sex-biased dispersal by influencing the costs and benefits of philopatric versus dispersive behaviours [8, 16]. Studies investigating sex-biased dispersal in fishes have so far produced examples of both male-biased (e.g., brook trout, *Salvelinus fontinalis*, [29]; *Neolamprologus pulcher*, [30]; three-spined sticklebacks, *Gasterosteus aculeatus*, [31]) and female-biased dispersal (e.g., Asian Seabass, *Lates calcarifer*, [32]). The variation that exists across species in terms of whether males or females are the more dispersing sex is particularly well-represented within fish family Cichlidae, which is also well-regarded for its extreme cross-species variation in social traits [33, 34]. Here, male-biased dispersal has been detected in rock- and sand-dwelling cichlids of Lake Malawi *Pseudotropheus* spp. [35] and *Copadichromis* spp. [36] as well as in *N. pulcher* from Lake Tanganyika [30]. No evidence of sex biased dispersal has been found in the Central American species, *Amphilophus astorquii* [37], and female-biased dispersal has been uncovered in some Tanganyikan species, such as *Eretmodus cyanostictus* [38], *N. caudopunctatus* [39], and *N. obscurus* [40]. It is interesting to note that dispersal biases in favour of one sex can be observed in cichlid species that also exhibit vastly different mating systems and breeding strategies. For example, *E. cyanostictus* is a biparental mouth brooder [38], *N. caudopunctatus* is a shoaling, but also biparental substrate spawner [39], and *N. obscurus* is a group-living, cooperative breeder [40], but all species display female-biased dispersal. The finding so far that neither male-biased nor female-biased dispersal is noticeably more prevalent within the cichlids stands in contrast to the more consistent patterns seen in other taxa, such as male-biased dispersal in mammals and female-biased dispersal in birds (see [8]), which makes them an intriguing system in which study sex-biased dispersal and its links to social evolution.

In this study, we tested for sex-biased dispersal as well as for environmental constraints on dispersal in a wild population of *Neolamprologus multifasciatus*, a group-living, highly social Lamprologine cichlid fish. *Neolamprologus multifasciatus* is among the smallest cichlid species endemic to Lake Tanganyika, East Africa, with males and females rarely exceeding 3.0 cm and 2.1 cm in standard length respectively in the wild [41, 42]. *Neolamprologus multifasciatus* can be found living in groups of up to ~ 20 individuals [34], and these groups consist of a single dominant male, several adult females and subordinate males, as well as immature, juvenile individuals [41–44]. Social groups of *N. multifasciatus* hold territories on vast regions of the lake floor called ‘shell beds’ where accumulations of empty gastropod shells cover the ground and the fish excavate these shells from the sandy substrata. The fish use these shells as shelters for evading predators and as brood chambers for females to raise their offspring. Over hundreds of hours of behavioural studies in the field, we have never observed clear instances of alloparental care in *N. multifasciatus* (AB, LK, AJ personal observations). Dispersal in *N. multifasciatus* appears to be delayed until individuals near or reach sexual maturity and adults can sometimes switch their group memberships between broods [42]. Thus, it is possible that *N. multifasciatus* movement patterns are comprised of both natal and breeding dispersal. While males and females may move between groups over their lifetimes, large males can also establish new territories on their own when space is available to them [42, 44]. *Neolamprologus multifasciatus* territories can be extremely densely distributed across the shell bed, sometimes with only ~ 30 cm separating nearest neighbouring territories [42, 44]. Therefore, in certain areas of the shell bed there may be ample alternative groups nearby to serve as potential dispersal destinations, whereas in other areas of the shell bed such options can be scarcer. Furthermore, dispersal distances are typically very short—often no more than 200 cm—as gauged by the spatial separation between parents and their offspring when living apart in the wild [42]. Such a limited scale of movement is likely due to a high risk of predation from piscivorous fishes that share the same habitat space [41].

*Neolamprologus multfasciatus* experience a number of ecological and social conditions that led us to predict dispersal would be sex-biased in this species, and that females might be the more-dispersing sex. To begin, we expected both sexes to be similarly constrained in their ability to disperse between territories on the shell bed as both males and females are susceptible to predation when moving away from their territories [41], though body size differences between the sexes might lead one sex to be more susceptible to predation than the other. Most reproduction is secured by the dominant male in their groups, which means that small, subordinate males achieve very little reproduction, both at home and in neighbouring territories [42]. Subordinate males are also unable to establish independent territories of their own until they have grown large enough and sufficient space is available nearby [42]. Thus, while subordinate males suffer low reproductive success at home, most of them also have few prospects for better success elsewhere. This might result in males remaining philopatric where they can queue for a breeding position. Relatedness between males can then promote cooperation and tolerance between cohabiting dominant and subordinate males. Unlike males, however, cohabiting females can reproduce concurrently [42], suggesting that young females have better prospects to reproduce soon after they emigrate than young males. Females should therefore be less likely to form queues in their natal groups, because the do have reproductive options elsewhere. Females are also highly competitive and will engage in agonistic interactions over limited resources within their groups, especially when offspring are being cared for [45]. This suggests little scope for cooperation among females, and implies low or negligible inclusive fitness benefits for female relatives that reside near to one another. Lastly, while both sexes experience aggressive resistance from resident fish when attempting to join a new group, potential male joiners receive more aggression overall than potential female joiners [46], which suggests that group switching may be costlier for males than for females.

In this study, we evaluated the hypothesis that dispersal is female-biased in *N. multifasciatus* by using pairwise relatedness estimates from extensive microsatellite genotyping of a wild population along with high-resolution spatial data. In total, we sampled 812 fish from 128 social groups. We assessed the relatedness structure of social groups and also examined how relatedness among individuals changes with increasing spatial separation across the neighbourhood; in doing so, we tested for sex differences in dispersal probabilities and dispersal distances [47]. Furthermore, if the dispersal potential of individuals is influenced by the distribution and availability of suitable territories to which to emigrate, then we also predicted to see higher within-group relatedness in groups from sparse regions of the shell bed relative to groups living in denser regions.

## Results

**Fig. 1:**
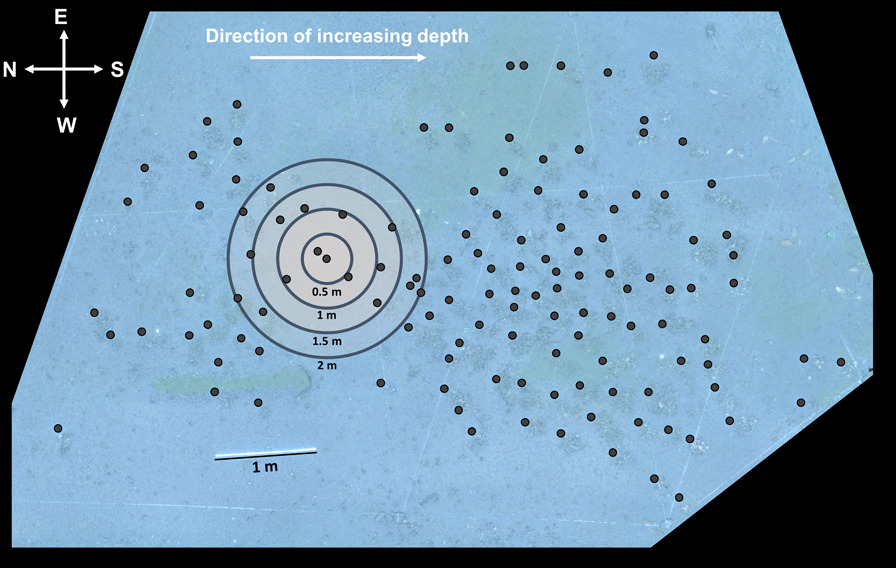
Map of the study quadrat at a depth of 10–11 m. Black dots indicate the positions of *Neolamprologus multifasciatus* territories. Note that this cluster of *N. multifasciatus* territories was surrounded by a stretch of bare sand, at least one meter in width, separating it from the rest of the shell bed. Radiating circles represent radii of 0.5, 1, 1.5, and 2 m around an example territory, visualizing how we calculated neighbourhood density (see “Methods” section). Approximate cardinal directions and a measure of scale are indicated on the map

**Fig. 2:**
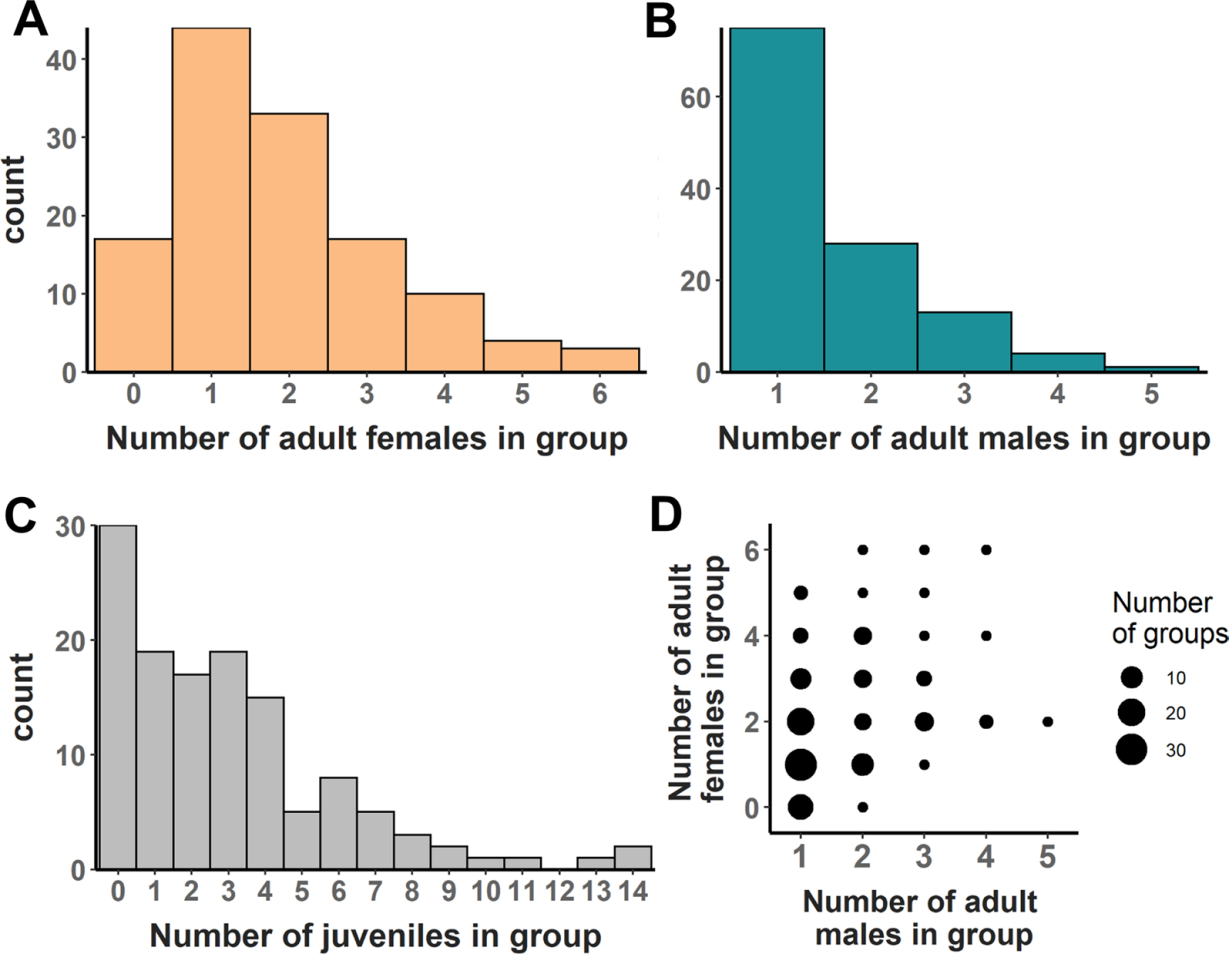
Histograms illustrating the number of **A** adult females, **B** adult males, and **C** juveniles within each *Neolamprologus multifasciatus* territory in our study quadrat. **D** Scatterplot showing the numbers of adult males and females in groups, where dot size scales with the number of groups found with the corresponding composition

All 128 N*. multifasciatus* territories that we sampled from our study quadrat in the wild (Fig. 1) contained a dominant male, though we were unable to capture seven of them for measurement and fin clipping. Each group contained on average (± SD) 6.3 ± 4.4 fish, which comprised 1.9 ± 1.4 females (range = 0–6, Fig. 2A), 1.5 ± 0.9 males (range = 1–5, Fig. 2B), and 3.0 ± 3.0 juveniles (range = 0–14, Fig. 2C). The most common sex composition was a single dominant male with one adult female (Fig. 2D). Groups containing more males also contained more females (Spearman correlation coefficient = 0.38).

### High within-group relatedness among males, but not among females

**Fig. 3:**
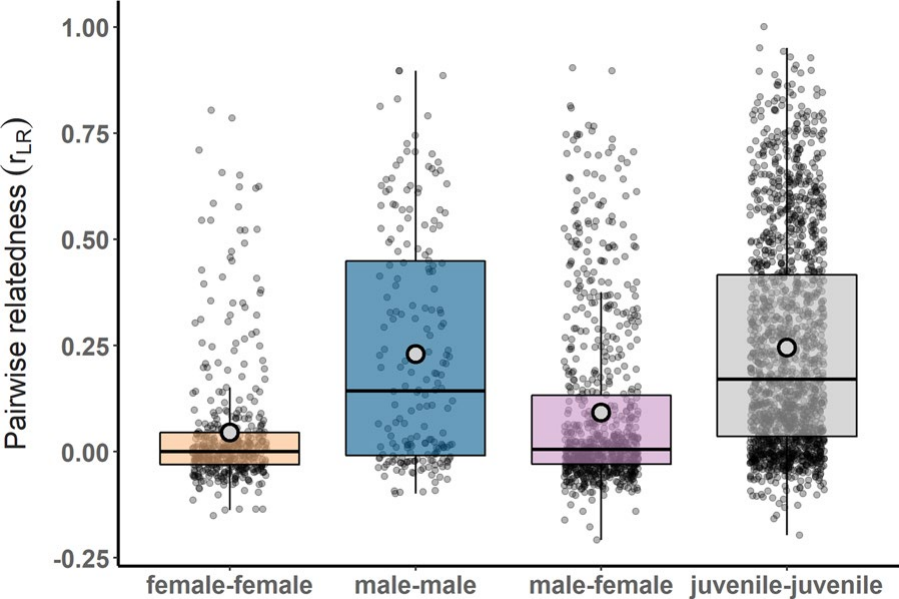
Pairwise relatedness estimates (r_LR_) for cohabiting *Neolamprologus multifasciatus* group members divided by sex and life stage pairing. Box plots indicate sample means (open circles), sample medians (horizontal lines), first and third quartiles (boxes), and the range of data within 1.5 interquartile distances (whiskers)

We calculated pairwise Lynch-Ritland estimators (r_LR_, [48]) to assess relatedness among the fish in our study. Females had low relatedness to other females in their groups, with an average (± SD) r_LR_ of 0.045 ± 0.15 (Fig. 3). Males were more related to each other within their groups (average (± SD) r_LR_ of 0.23 ± 0.27) than females were to each other (LMM, Est. ± SE = 0.16 ± 0.027, z = 6.08, *P* < 0.001). Adult males and females living in the same groups had average relatedness estimates of 0.092 ± 0.20 to each other, while juveniles were related to other juveniles in their groups at a level of 0.24 ± 0.24. Table 1 summarizes all Tukey contrasts between cohabiting fish types.

**Table 1:** Statistical output of linear mixed effects model examining within-group relatedness among adult males, adult females, and juveniles in *Neolamprologus multifasciatus* groups

Contrast	Estimate ± SE	z-value	*P*-value
Male-male vs. female-female	0.16 ± 0.03	6.08	< 0.001
Female-male vs. female-female	0.034 ± 0.018	1.91	0.22
Juvenile-juvenile vs. female-female	0.21 ± 0.02	12.2	< 0.001
Female-male vs. male-male	− 0.13 ± 0.02	− 5.33	< 0.001
Juvenile-juvenile vs. male-male	0.049 ± 0.024	2.02	0.17
Juvenile-juvenile vs. female-male	0.18 ± 0.01	12.3	< 0.001

All pairwise comparisons were made using the Tukey method in the “multcomp” R package [49]

### Relatedness among males between groups falls more steeply with spatial separation than for females

**Fig. 4:**
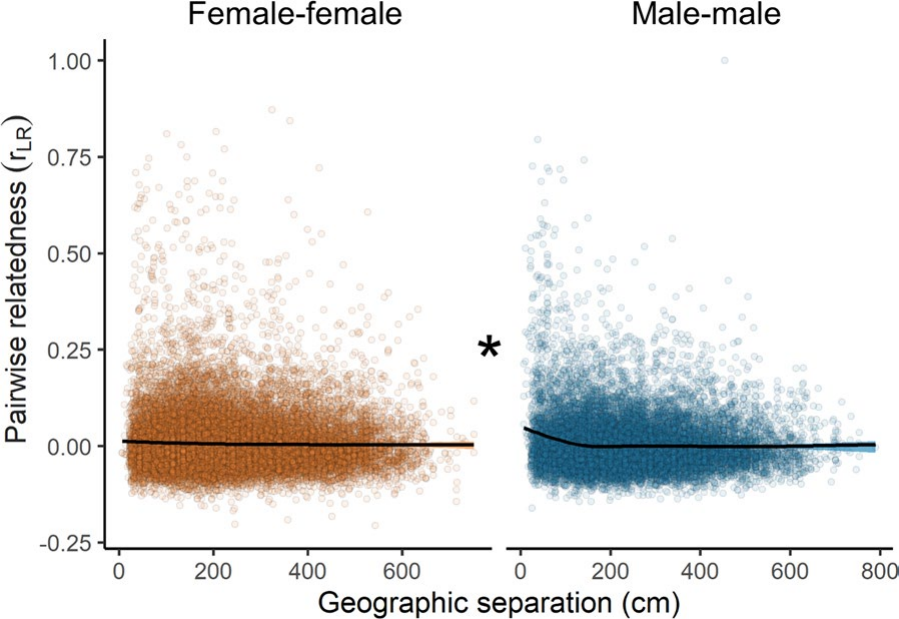
Relatedness among between-group, same-sex adults declines with geographic separation. A stronger decline is evident for male-male relatedness than for female-female relatedness, particularly across the first two meters around each individual. Plots show GAM fits built with cubic regression splines (see “Methods” section). This plot shows raw, untransformed data

Female-female relatedness between groups declined with geographic separation (GAM, edf = 2.43, F = 8.49, *P* < 0.0001), though the slope was very shallow (Fig. 4). Male-male relatedness, on the other hand, declined more steeply with geographic separation than female-female relatedness (GAM, edf = 3.91, F = 36.6, *P* < 0.0001), and this sex difference was primarily seen within a two-meter radius around focal individuals (Fig. 4).

### Female dispersal, but not male dispersal, is influenced by neighbourhood density

**Fig. 5:**
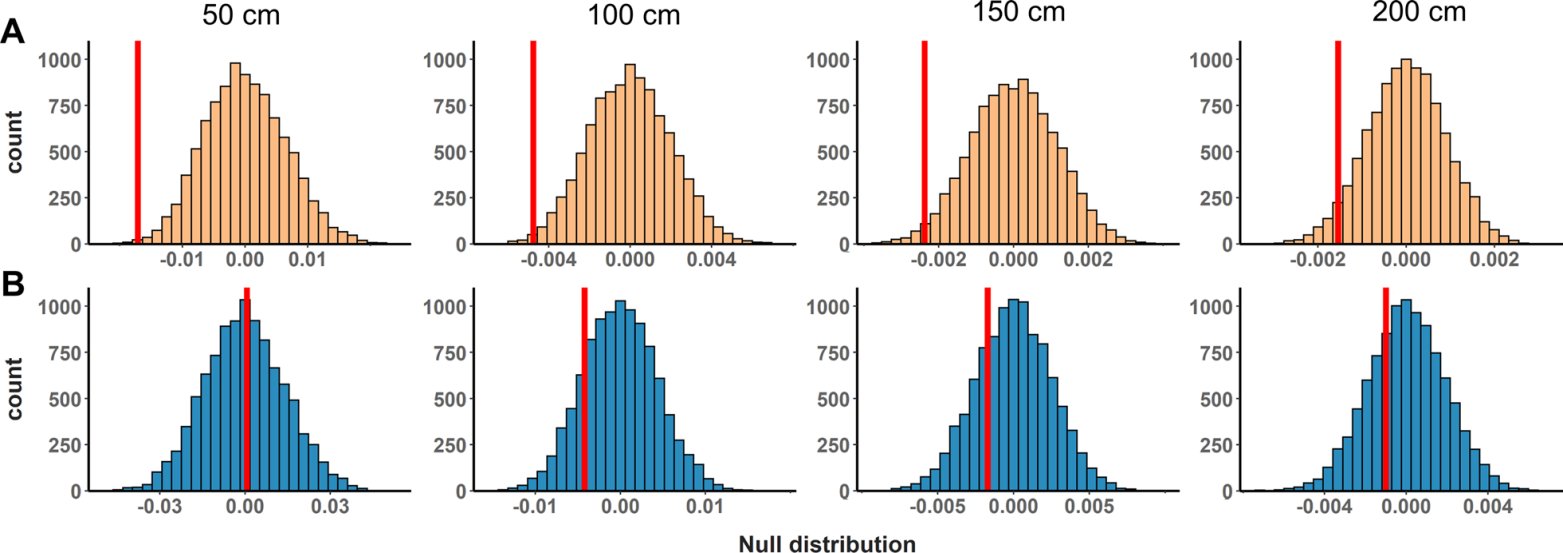
Histograms showing null distributions from permutation tests described in Methods. Null distributions represent slopes from a regression between average same-sex, within-group relatedness and the number of neighbouring *Neolamprologus multifasciatus* territories within a radius of 50 cm, 100 cm, 150 cm, and 200 cm. Vertical red lines indicate the observed slopes. **A** Results from female-female analyses. **B** Results from male-male analyses

We calculated neighbourhood densities at multiple spatial scales by counting the number of other *N. multifasciatus* territories within 50 cm, 100 cm, 150 cm, and 200 cm of each focal group (Fig. 1). Then, using permutation tests, we found that within-group female-female relatedness was higher when there were few neighbouring territories, and was lower when there were many neighbouring territories. This pattern was evident at all spatial scales tested: 50 cm (*P* = 0.0019), 100 cm (*P* = 0.007), 150 cm (*P* = 0.018), and 200 cm (*P* = 0.043, Fig. 5A). In contrast, within-group male-male relatedness was unrelated to the number of neighbouring territories at all spatial scales: 50 cm (*P* = 0.51), 100 cm (*P* = 0.16), 150 cm (*P* = 0.25), and 200 cm (*P* = 0.30, Fig. 5B).

## Discussion

In this study, we uncovered evidence of female-biased dispersal in *Neolamprologus multifasciatus*, a highly social, group-living cichlid fish. Estimates of relatedness among cohabiting females were low, especially when compared to males, who shared a higher level of relatedness to other males in their groups (r_LR_ = 0.23 ± 0.27, average ± SD, for males compared to 0.045 ± 0.15 for females). Furthermore, average relatedness coefficients among females from different territories were low, and this was independent of whether females resided in closely neighbouring or more distant territories. In contrast, males displayed elevated relatedness to their male neighbours relative to males living farther away. There was a distinct pattern of decreasing relatedness with geographic separation among males within our study quadrat, which was discernable over a spatial scale of approximately two meters. Taken together, these data indicate female-biased dispersal within our study system both in terms of probability for females to emigrate from their groups, as revealed by within-group relatedness patterns, and the distances over which females travel, as revealed by between-group relatedness patterns.

Our results indicate that the social environments within *N. multifasciatus* groups are characterized by a marked asymmetry in how related males and females are to their same-sex group mates. While males tended to be related to other males in their group, females were on average unrelated to other females (here, relatedness was computed with reference to the whole study quadrat). The question now, however, is whether such relatedness structure within groups coincides with patterns of sex-specific behaviour observed in our study species. Low average relatedness among females is consistent with the findings of previous studies that have documented high female-female conflict in this species [43, 45]; however, competition for resources and mates in *N. multifasciatus* is also thought to occur on extremely local spatial scales, which can lead to competition among individuals that is less affected, or even unaffected, by their relatedness [11]. In *N. multifasciatus*, females divide the territory space into discrete sub-territories that they defend from each other, a behavioural pattern that is thought to be the outcome of intrasexual competition [45, 46]. Interactions among cohabiting males have been relatively unstudied to date, however, dominant males can clearly tolerate subordinate males to live on their territories. Given that dominant males are larger and more competitive than subordinates and that subordinates are often limited in their ability to establish their own independent breeding groups [42], this means that subordinates are ill-equipped to compete for reproduction at home or to move elsewhere, and may therefore be forced to make the best-of-a-bad-job by queuing to inherit an eventual breeding position. Tolerance of subordinate males forming such queues can be facilitated by genetic relatedness and/or by subordinates offering direct fitness benefits to the dominant male (i.e., pay-to-stay, [50, 51]). Indeed, we found that groups with additional males also supported more females (Fig. 2D), suggesting that subordinate males might provide direct fitness benefits to the dominant male by helping to maintain a larger and more productive territory. Field observations indicate that subordinate males, along with females, will contribute to territory defense, especially when intruders approach their sub-territories [41, 46]. We therefore suggest that while females disperse more often and move further afield, males will generally remain on their natal territories as subordinates and either inherit a future dominant breeding position, or move to a nearby vacancy when one becomes accessible.

Numerous proximate factors are commonly invoked to explain sex-biases in dispersal, and they include inbreeding avoidance (19–22; but see [16, 52]), asymmetric handicaps [8, 16, 23] and local mate or resource competition [17–19]. Female-biased dispersal in *N. multifasciatus* supports two of these non-mutually exclusive hypotheses. Since *N. multifasciatus* groups represent polygynous harems in which females have a single mating partner (i.e., the dominant male), while the dominant male may have multiple partners, the inbreeding avoidance hypothesis would predict that females should be more likely to disperse in order to avoid incestuous matings comprising a large proportion of their lifetime reproduction (but see [16, 52, 53]). Males also experience a significant handicap relative to females when attempting to switch groups because they are met with intense aggression from resident fish when acting as prospective joiners [46]. *Neolamprologus multifasciatus* also express sexual dimorphism with males being larger than females [41]. This raises the possibility that large body sizes impose higher dispersal handicaps than small body sizes in this system. For example, larger bodied fish may find it more difficult to seek shelter when venturing away from their own shells, perhaps due to the relative scarcity of large gastropod shells [54] into which they fit. This remains an open question for future research in *N. multifasciatus* and other shell-dwelling cichlids. Males experiencing higher dispersal costs than females have been detected in other taxa as well including the Seychelles warbler, *Acrocephalus sechellensis*, where males suffer higher mortality rates during extra-territorial forays than females [15]. Thus, multiple factors may be working jointly to promote female-biased dispersal in *N. multifasciatus*, and future work will be needed to tease apart their relative contributions. Interestingly, the local mate or resource competition hypothesis [17–19] predicts male-biased dispersal in N*. multifasciatus*, because while both sexes engage in within-group competition over reproduction, reproductive skew is much higher among same-group males than females [42]. However, as mentioned above, most subordinate males have poor options for establishing new territories of their own or being allowed to settle on a neighbouring territory, and so they cannot readily escape their local competitive environment.

Though females are the more-dispersing sex in *N. multifasciatus*, we also detected a marked constraint on their dispersal, namely the availability of nearby groups that might allow them to join. The shell-beds of Lake Tanganyika can be extremely densely packed with shell-dwelling cichlids [34], and our study population displays an average of ~ 30 cm separating nearest neighbouring *N. multifasciatus* territories [42, 44]. While certain regions of the shell bed offer a dispersing fish numerous destination options nearby, other regions can be more sparsely populated. We found that the more neighbouring territories there were within a reachable distance (i.e., tested up to two meters), the less related females were to their other female group-mates—a pattern that was not detected among males. Such a sex difference might arise because males are more philopatric to begin with, but also because males can establish their own territories, or forcefully take over neighbouring territories if they have grown large enough [42]. Another, non-mutually exclusive, explanation is that in sparser regions of the shell bed, females may be discouraged from dispersing due to wider average distances of open sand separating territories acting as a movement barrier. Our results highlight the importance of accounting for the spatial distribution of suitable dispersal destinations in shaping the movement patterns of individuals. This is in agreement with previous studies that have similarly emphasized the role of dispersal site availability (e.g., great tits, *Parus major*, [55, 56]; lesser kestrels, *Falco naumanni*, [5]).

In conclusion, we present evidence of female-biased dispersal in the group-living and highly social cichlid *N. multifasciatus*, and also suggest that the spatial distribution and availability of nearby territories on which to settle represents an important ecological constraint on their movement. The genetic structure of the social environment of *N. multifasciatus* is highly asymmetrical, with most cohabiting females being unrelated, and most males sharing a significantly higher degree of relatedness to each other. This asymmetry in relatedness among males and females means that their social behaviour will likely generate complex fitness costs and benefits, which can in turn influence selection on decisions of when and where to disperse in an evolutionary feedback loop [2]. Cichlid fishes showcase a great diversity of mating systems, parental care strategies [57], and social structures [34, 58] and we suggest that they can also be a highly valuable system for gaining new insights into the evolution and expression of sex-biased dispersal and its relationship with social evolution.

## Methods

### Study site and field sampling

Our study site was located in a large shell-bed on the lake floor at the southern tip of Lake Tanganyika, Zambia (8°42′49.0″ S 31°07′22.9″ E). Between September and October 2019, we identified all *N. multifasciatus* territories in a study quadrat on the shell bed (quadrat size measured approximately 10 × 10 m and was located at a depth of 10–11 m). Our study quadrat enclosed a cluster of territories that were separated from the rest of the shell bed by a border of open sand that was at least one meter wide. While on SCUBA, we took downward facing video footage of the entire study quadrat (GoPro Hero 7 camera set to 1080p resolution, 30 fps, and a ‘linear’ field of view) and used Structure-from-Motion photogrammetry [59, 60] to recreate the spatial layout of the territories in the study area (Fig. 1). From this spatial layout, we calculated the pairwise distances between all *N. multifasciatus* territories based on their Cartesian coordinates as placed in ImageJ (v 1.53e). No territories of any other shell-dwelling Lamprologine cichlids (see [34]) were present within this study quadrat. A buddy pair of two divers systematically sampled all individuals that could be captured from each territory. Capturing *N. multifasciatus* individuals can be done by hand as the fish hide within shells when approached by predators or SCUBA divers. While underwater, the shells containing hiding fish were picked up and the fish were extracted from their shells and sedated with clove oil. We then sexed each fish by inspecting their urogenital papillae, measured them for standard length (cm, SL), and recorded them as either an adult or a juvenile based on the presence of dark banding patterns along the sides of their bodies, which denote sexual maturity [41]. Social groups were defined as the fish living in close proximity to one another, often all within a single crater excavated from the sand on the lake floor containing a collection of empty gastropod shells. These groups are also easily identified as they are physically separated from neighbouring craters constituting other *N. multifasciatus* territories. Fish within a social group would also share abutting or overlapping home ranges and frequently interact with one another [45]. We recorded the largest male in each group as the dominant male and any further males within the group as subordinate males [41, 42]. We fin-clipped large fish (> 1.7 cm in SL) on their anal fins (taking at most 2 × 2 mm of tissue). When the fish had fully recovered from sedation, we returned them and their shells to their original territories. Any fish smaller than 1.7 cm (~ 20% of our whole sample) were euthanized with an overdose of clove oil and sampled whole because of the relatively large amount of tissue that fin-clipping would have removed. All tissue samples and whole fish were stored in 99% ethanol, and transported back to the lab for later microsatellite genotyping. To ensure that we had captured as many of the fish from each group as possible, we returned to each territory on at least two further occasions to check for unclipped fish. Any missed fish were similarly captured, sexed, measured, and fin-clipped. After two consecutive visits to each territory, unclipped fish were exceptionally rare, lending high confidence that we had sampled all or nearly all of the individuals living in the study quadrat. In total, we sampled 812 fish (239 adult females, 191 adult males, and 382 juveniles) from 128 territories, which constituted all territories in the study area.

### Microsatellite genotyping and marker polymorphism

The microsatellite data obtained from the fish in this study quadrat were also used in other studies (e.g., 42). In the lab, DNA was extracted from the tissues using a standard Chelex protocol [61]. All individuals were genotyped at 20 microsatellite loci divided into three multiplexes (Table 2). We used 3 µL of Qiagen Type-it Multiplex PCR Master Mix for the multiplex PCRs, along with 1 µL of template DNA, and 0.5 µL of primer mix (see Table 2 for concentrations). Total PCR volume was 5.5 µL, and each forward primer was labeled with one of the fluorescent dyes HEX, FAM, NED, ATTO550 and ATTO565. We used the following PCR program settings: denaturation at 95 °C for 5 min, followed by 30 cycles at 95 °C for 30 s, annealing at 55 °C (for multiplex1), 54 °C (for multiplex 2), or 53 °C (for multiplex 3) for 90 s, extension at 72 °C for 30 s, and a final extension at 60 °C for 30 min. We scored allele sizes against an internal standard (GeneScan 500 LIZ, Applied Biosystems) in an automatic sequencer (3130xL Genetic Analyzer, Applied Biosystems) and GeneMapper software (v 3.7, Applied Biosystems).

**Table 2:** Marker polymorphism of 20 microsatellites used in this study based on reference population

Locus	k	N	H_Obs_	H_Exp_	HW *P*-value	Conc. in primer mix (pmol/μL)	References
*Multiplex 1*							
Pmv17	19	233	0.906	0.912	0.50	0.5	[63]
UNH890	6	232	0.414	0.436	0.73	1.0	[64]
UNH908	25	235	0.843	0.875	0.36	3.0	[64]
Gm634	15	234	0.799	0.818	0.42	1.0	[65]
Ppun9	21	233	0.674	0.748	0.02	0.5	[66]
Hchi59	17	232	0.845	0.864	0.52	1.0	[67]
UNH216	11	232	0.603	0.584	0.83	4.0	[68]
UME002	7	228	0.61	0.627	0.44	4.0	[69]
*Multiplex 2*							
Pmv3	31	237	0.768	0.775	0.04	1.0	[63]
GM264	17	234	0.85	0.859	0.42	4.0	[65]
Ppun5	23	233	0.695	0.722	0.19	3.0	[66]
TmoM13	25	234	0.829	0.907	0.44	4.0	[70]
TmoM25	4	231	0.732	0.671	0.55	2.0	[70]
Hchi36	4	230	0.539	0.559	0.44	1.0	[67]
UME003	17	232	0.897	0.869	0.56	2.0	[69]
*Multiplex 3*							
TmoM11	7	234	0.667	0.677	0.87	1.5	[70]
UNH2075	19	233	0.773	0.77	0.23	2.5	[71]
NP101	18	232	0.81	0.749	0.02	3.5	[72]
Pzeb4	8	232	0.612	0.61	0.98	2.0	[73]
UNH974	33	219	0.863	0.926	0.60	4.0	[64]

k: Number of alleles, N: Number of individuals genotyped at the particular locus, H_Obs_: Observed Heterozygosity (proportion of heterozygotes at this locus), H_Exp_: Expected heterozygosity (expected proportion of heterozygotes given allele frequencies), HW: Adherence to Hardy–Weinberg Equilibrium, tested in Cervus using a Bonferroni correction (Bonferroni corrected α = 0.0025)

We estimated population allele frequencies in CERVUS (v 3.0.7; [62]), using a subset of fish sampled from the quadrat. To reduce the influence of within-group kinship structure, we chose the dominant male and up to one random female from each territory (N = 233 fish). The markers were highly polymorphic with an average of 16.4 alleles per locus, a mean heterozygosity of 0.75, and all markers adhered to Hardy–Weinberg equilibrium (Table 2).

### Relatedness estimates

All analyses in this study were carried in out in R [74]. We used the R package “Demerelate” [75] to calculate symmetric Lynch-Ritland pairwise relatedness estimators (r_LR_, [48]) for each pair of fish in our dataset that had at least 10 microsatellite loci successfully genotyped (772 out of 812 or 95.1% of all individuals sampled, which was 97.9% of all adult females, 99.0% of all adult males, and 91.4% of all juveniles). We chose r_LR_ as an estimator because it performs well for weakly related or unrelated individuals [76–78], which we expected to constitute the majority of cases in our dataset.

### Statistical analyses

We used the R package “glmmTMB” [79] to fit all linear mixed effects models (LMMs), and inspected model diagnostics using the R packages “performance” [80] and “Dharma” [81]. We used the R package “mgcv” [82] to fit our generalized additive models (GAMs).

### Group composition

We first examined the sex and life stage compositions of our *N. multifasciatus* groups, by generating frequency histograms reflecting the number of males, females, and juveniles comprising each group. We also related the number of males residing on each territory to the number of females using a linear regression model (LM).

### Is sex-biased dispersal revealed by the relatedness structure of *N. multifasciatus* groups?

We now asked whether pairwise relatedness estimates (r_LR_) differed among cohabiting females, males, and juveniles. We assembled all r_LR_ corresponding to pairs of fish living in the same groups as each other and examined the female-female, male-male, male–female, and juvenile-juvenile pairings (N = 1514 fish pairings living in 109 groups). Note that some groups were not able to contribute relatedness estimates for all pairings; for example, if a group contained only one female then we could not calculate a within-group estimate of female-female relatedness. Furthermore, if a territory was occupied by a solitary male, then this male could only contribute to between-group relatedness estimates (see below). We fit a LMM to the r_LR_ estimates and included ‘fish pairing’ as a predictor variable (4-level categorical). Note that each r_LR_ value corresponds to a pair of fish—Fish 1 and Fish 2—yet we opted not to include the identities of Fish 1 and Fish 2 as random effects because it was not possible to structure the random effects such that the intra- and inter-individual variation attributable to fish identities could be accounted for. Instead, we included a random intercept of ‘Group ID’. All pairwise contrasts between the levels of ‘fish pairing’ were then tested for statistical significance with the “multcomp” R package [49] using the Tukey method.

### How does relatedness change with geographic separation between individuals?

We examined how male-male and female-female relatedness changed with geographic distance between groups. Here, we assembled all r_LR_ corresponding to pairs of same-sex adult fish living in different groups (N = 44,246 fish pairings across all 128 territories). We used a generalized additive model (GAM) assuming a Gaussian error distribution to accommodate any potential non-linear relationships between r_LR_ and the geographic distance between groups. We first applied a Yeo-Johnson power transformation to the r_LR_ values to improve the normality and symmetry of the model residuals [83]. We included ‘fish pairing’ as a predictor variable (2-level categorical: male-male vs female-female), as well as geographic distance between groups (in cm) as a cubic regression spline composed of five knots. We also looked for a sex difference in the potentially non-linear relationship between geographic distance and r_LR_.

### Does neighbourhood density influence dispersal patterns?

We tested whether within-group relatedness among same-sex individuals varied with the number of territories in the nearby environment. For each *N. multifasciatus* territory in our quadrat, we calculated the number of other territories within radii of 50 cm, 100 cm, 150 cm, and 200 cm (Fig. 1). When not living in the same groups as their offspring, the vast majority of *N. multifasciatus* parents can be found living within 200 cm of their progeny [42] and so this represents an ecologically relevant spatial scale over which to look for dispersal patterns. We fit a series of linear regression models, one for each radius tested. We included average within-group female-female r_LR_ as the response variable, and the number of neighbouring territories that each group had within their radius as a predictor variable. We then repeated this using average within-group male-male r_LR_ values. Note that the female-female analyses only considered multi-female groups (*N* = 65), and the male-male analyses only used multi-male groups (*N* = 46). We applied a permutation-based approach to calculate *P*-values in which we ran 10,000 permutations of each model randomizing the response variable without replacement. *P*-values were calculated as the proportion of randomizations yielding slope values for the relationship between average within-group r_LR_ and neighbourhood density that were more extreme (i.e., more negative) than our observed slopes.

## Supplementary Information

**Additional file 1.** Between-group data. Pairwise Lynch-Ritland relatedness estimates for each pairing of fish that reside in different social groups from one another. Dataset also includes the spatial distances separating social groups (measured in cm).

**Additional file 2.** R script. R script to replicate all analyses in this paper.

**Additional file 3.** Group density data. Dataset containing the number of social groups found near each focal group (neighbourhood density as measured over 50 cm, 100 cm, 150 cm, and 200 cm). Dataset also contains the average within-group relatedness estimates (pairwise Lynch-Ritland) for both male-male and female-female comparisons.

**Additional file 4.** Within-group data. Pairwise Lynch-Ritland relatedness estimates for each pairing of fish that reside together in the same social group.

## Data Availability

All analyses can be reproduced with the data and R code provided in Additional files 1, 2, 3, 4.
